# Three-Dimensional Transesophageal Echocardiography as an Alternative to Multidetector Computed Tomography in Aortic Annular Diameter Measurements for Transcatheter Aortic Valve Implantation

**DOI:** 10.3390/biology10020132

**Published:** 2021-02-08

**Authors:** Ciprian Nicusor Dima, Marian Gaspar, Cristian Mornos, Aniko Mornos, Petru Deutsch, Horia Cioloca, Simona Cerbu, Mihai Dinu, Bogdan Hoinoiu, Constantin Tudor Luca, Lucian Petrescu

**Affiliations:** 1 Discipline of Cardiovascular Surgery, “Victor Babeş” University of Medicine and Pharmacy, Eftimie Murgu Sq. No. 2, 300041 Timișoara, Romania; dima_ciprian97@yahoo.com (C.N.D.); gaspar.marian@umft.ro (M.G.); 2 Institute of Cardiovascular Diseases, Gheorghe Adam St., No. 13A, 300310 Timișoara, Romania; cmornos@cardiologie.ro (C.M.); mornosaniko@yahoo.com (A.M.); deutsch.petru@umft.ro (P.D.); horiacioloca@yahoo.com (H.C.); ctluca@cardiologie.ro (C.T.L.); petrescu.lucian@umft.ro (L.P.); 3 Discipline of Cardiology, “Victor Babeş” University of Medicine and Pharmacy, Eftimie Murgu Sq. No. 2, 300041 Timişoara, Romania; 4 Department of Surgery, “Victor Babeş” University of Medicine and Pharmacy, Eftimie Murgu Sq. No. 2, 300041 Timişoara, Romania; 5 Discipline of Radiology and Medical Imaging, “Victor Babeş” University of Medicine and Pharmacy, Eftimie Murgu Sq. No. 2, 300041 Timișoara, Romania; 6 Faculty of Medical Engineering, University “Politehnica” of Bucharest, Gheorghe Polizu St., No. 1-7, 011061 Bucharest, Romania; mihaidinu88@gmail.com; 7 Department of Clinical Practical Skills, “Victor Babeş” University of Medicine and Pharmacy, Eftimie Murgu Sq. No. 2, 300041 Timişoara, Romania

**Keywords:** aortic stenosis, transcatheter aortic valve implantation, imaging, multi-detector computer tomography, three-dimensional transesophageal echocardiography

## Abstract

**Simple Summary:**

Patients who have multiple associated comorbidities and need to change the aortic valve may have a contraindication to open-heart surgery, the alternative being transcatheter aortic valve implantation, which requires very precise measurements of the aortic annulus to determine the dimensions of the prostheses. Ultrasonographic imaging techniques, such as transesophageal echocardiography, are constantly evolving. The aim of our study was to compare the three-dimensional transesophageal echocardiography and multi-detector computer tomography methods, with the former being an alternative for patients who cannot undergo computer tomography because of a major contraindication. We have demonstrated that there were small differences between aortic annular measurements using multi-detector computer tomography (2.25 ± 0.19 cm) and three-dimensional transesophageal echocardiography (2.25 ± 0.15 cm). Thus, three-dimensional transesophageal echocardiography can be the solution for aortic annular measurements used to select the correct prosthesis for the transcatheter aortic valve implantation procedure in patients who cannot undergo computer tomography.

**Abstract:**

*Background and objectives:* Transcatheter aortic valve implantation (TAVI) is a therapeutic choice for high surgical risk patients, serving as an alternative to open-heart surgery. Correct measurement of the aortic annulus, which leads to the selection of a suitable prosthesis and accurate outcome prediction, is essential for the success of TAVI. The objective of this study is to evaluate the accuracy of novel imaging te chniques in measuring the aortic annulus by comparing multi-detector computer tomography (MDCT) and three-dimensional transesophageal echocardiography (3D TEE) for the selection of the optimal prosthesis. *Materials and Methods:* Measurements of the aortic annulus have been performed on 25 patients using MDCT and TEE, and the correlation and agreement levels between the two measuring techniques were analyzed. MDCT measurements were used for the sizing of the prostheses. *Results:* MDCT and TEE measurements of aortic annular diameters were significantly correlated, with a mean difference of 0.001 cm. *Conclusions:* 3D TEE measurements have been in good agreement with MDCT and, therefore, 3D TEE can be used as an alternative in cases where MDCT is contraindicated or not available.

## 1. Introduction

In clinical practice, the precise measurement of the aortic annulus prior to transcatheter aortic valve implantation (TAVI) has a significant impact on procedural decisions and prosthetic valve sizing recommendations [1]. If the size of the prosthetic valve is larger or smaller than the aortic root, it can lead to various problems. Placing a valve that is larger than the optimal size can cause high deployment, prosthetic embolism, or paravalvular leakage, while deploying a smaller valve can cause trauma of the aortic root, ostial blockage, incorrect stent expansion, and diminished cusp mobility, or low deployment with either trans-stent leakage or impaired mitral valve function [2].

Multi-detector computer tomography (MDCT) has been shown to be an essential measuring technique prior to TAVI in order to minimize paravalvular leakage and is widely considered the gold standard for aortic annular assessment [3]. However, novel imaging techniques, such as three-dimensional transesophageal echocardiography (3D TEE), can provide similar accuracy to MDCT in aortic annular diameter measurement, as shown in a recent systematic review incorporating 19 studies that compared the two imaging techniques [4]. Moreover, it was shown that 3D TEE can be successfully deployed as an alternative to MDCT for pre-TAVI measuring of the aortic annulus in certain patients due to the lack of exposure to contrast agents.

In this study, 3D TEE and MDCT aortic annular diameter measurement accuracy were compared, with the aim of determining if 3D TEE can be a viable alternative to MDCT in preprocedural aortic annular diameter measurements for the sizing of prosthetic aortic valves produced from bovine and porcine pericardial tissue in addition to cobalt-chromium and nitinol frames, respectively, required for TAVI. This study represents an East European population group and can contribute to a major international study with the goal of improving the current knowledge on 3D TEE as a preprocedural measuring technique for TAVI.

## 2. Materials and Methods

### 2.1. Study Population

From January 2018 to January 2020, 25 high-risk patients with severe symptomatic AS, having an aortic valve area of 0.7–1 cm^2^, underwent TAVI at the Institute of Cardiovascular Diseases in Timisoara. Several patients had significant comorbidities, such as porcelain aorta, previous thorax irradiation, advanced age, obesity, pulmonary fibrosis, and hematological diseases. One of the patients who underwent TAVI had bicuspid aortic valve. The decision to proceed with TAVI was discussed by a heart team, including a cardiologist, an interventional cardiologist, anesthesiologists, and cardiovascular surgeons. All patients met the inclusion criteria; namely, being diagnosed with AS and/or being non-surgical patients. The exclusion criteria were the size of the aortic annulus not corresponding to any of the available prostheses and/or lack of access to the vascular route. Following the assessment of the vascular route using Doppler echocardiography for the femoral and iliac artery and 3D TEE for the descending aorta, no anomalies have been found in any of the 25 patients. All 25 patients have provided written informed consent. Approval has been granted by the local Ethics Committee.

### 2.2. TAVI: Procedure, Prosthetic Valves, and Aortic Annular Sizing

TAVI procedures were performed via the transfemoral approach, using balloon-expandable Edwards SAPIEN 3^TM^ valves (Edwards Lifesciences, Irvine, CA, USA) in 84% of cases and self-expandable Medtronic CoreValve prostheses (Medtronic, Minneapolis, MN, USA) in 16% of them. The SAPIEN valve is composed of bovine pericardial tissue attached to an expandable cobalt-chromium frame, while the CoreValve is composed of porcine pericardial tissue and has a self-expanding nitinol frame. In all cases, both preprocedural 3D TEE and MDCT were performed to measure the aortic annulus. Given the fact there is no consensus regarding the best imaging technique for aortic annular sizing, with several authors advocating a multi-modal approach [5,6], the valve manufacturer’s guidelines were followed, and MDCT data was used for sizing the prosthesis, detailed reconstruction of the aortic valve, and showing the calcific deposits. Similar to recent studies concerning TAVI sizing [7,8], this approach has been used to avoid discrepancies with the manufacturers’ sizing algorithms. TAVI procedures were performed on patients under general anesthesia in a catheterization laboratory with the aid of fluoroscopy and TEE imaging.

In absence of aortic annular and cusp calcifications, the TAVI procedure becomes extremely problematic due to the difficulty of anchoring and fastening the prosthetic valve. The absence of calcifications, aortic root dilatations, and increased systolic volume represent limitations in positioning and fastening the prosthetic valve, leading to complications such as prosthetic heart valve thrombosis or postprocedural paravalvular leakage [9]. Furthermore, the prosthetic valve can migrate to either the aorta or the left ventricle within a few hours after implantation, and, therefore, it has been proposed that oversizing the prosthetic valve by 15–20% can reduce this risk; however, oversizing the valve by more than 20% is not advised as it can lead to annular rupture [10,11].

### 2.3. 3D TEE

3D TEE measurements have been performed using the Philips iE33 ultrasound imaging system (Philips Medical Systems), equipped with the X7-2t multiplanar transesophageal probe. The 3DQ software (Q-Lab version 7, Philips Medical Systems) was used to process and analyze the images. Measurements of the aortic annulus were performed in the mid-oesophageal long-axis view. All patients underwent topical oropharyngeal anesthesia using 10% xylocaine. All TEE measurements were performed by an experienced cardiologist with over 15 years of experience in echocardiography, who was blinded to the MDCT measurements, which were performed by the valve manufacturers. An experienced heart team evaluated preoperative, perioperative, and postoperative TEE data.

### 2.4. MDCT

MDCT imaging was performed using a SOMATOM Sensation 64 Multi-Slice CT (MSCT) scanner (Siemens Medical Solutions) using the standard technical parameters, including automatic tube-current modulation, ECG gating, and field of view (FOV) limitation. An iodine-based contrast agent was used. Measurements and image analysis were performed by the prosthetic valve manufacturers, using their standard procedures and image processing software.

### 2.5. Statistical Analysis

The whole study was based on a cohort of 25 patients who satisfied the inclusion criteria. Continuous data are expressed as mean ± standard deviation (SD), while categorical data are expressed as numbers or percentages. The variables were analyzed with the χ^2^ test as appropriate. A Student’s *t*-test was used to compare 3D TEE and MDCT continuous variables. Analysis of variance (ANOVA) was used for multiple comparisons of aortic annular measurements. The Bland–Altman analysis was used to verify the bias and levels of agreement between the two methods. All *p*-values reported under 0.05 were considered statistically significant. Statistical analyses were performed using SPSS Version 26 (Chicago, IL, USA).

## 3. Results

### 3.1. Population

A population of 25 patients who underwent TAVI has been observed during a two-year reference period. Patients have been examined prior to the TAVI procedure using MDCT and 3D TEE in order to measure the aortic annular diameters, required for the sizing of the prosthetic valve replacement. All 25 patients have been selected for this study. The only selection criterium was the presence of both MDCT and 3D TEE aortic annular measurements. The male:female ratio was 14:11 (56% male patients, 44% female patients). The mean age at presentation was found at 76.32 ± 6.08 years, with the median and the mode corresponding both at the age of 78, showing a quite uniform distribution among age.

### 3.2. Measurements

**Figure 1:**
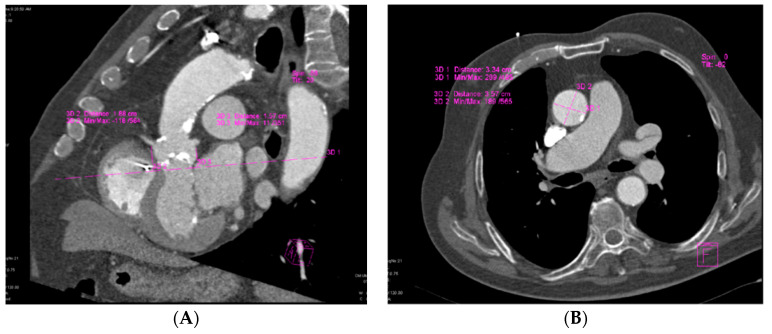
Assessment of aortic annular plane via MDCT: (**A**) Aortoventricular centerline; (**B**) Manual measurements of aortic annulus diameter.

MDCT, which offers good visualization of aortic valve anatomy and extensive calcifications [7], provided accurate measurements for aortic annular sizing. The size of the implanted prosthesis correlated significantly with the MDCT aortic annular measurements (Figure 1).

**Figure 2:**
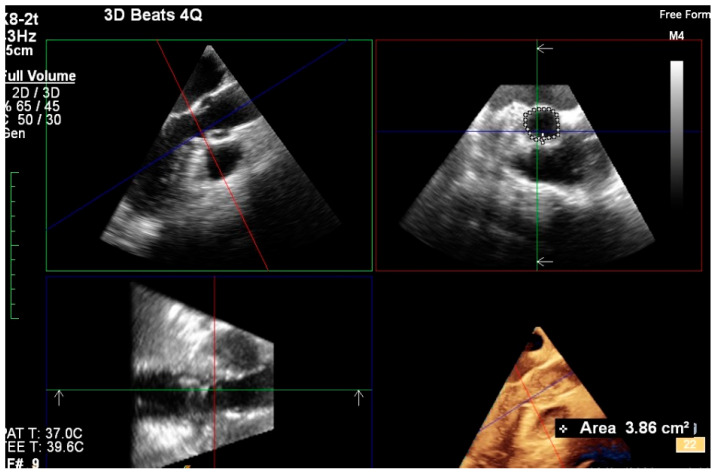
Assessment of aortic annular plane via 3D TEE imaging.

3D TEE imaging (Figure 2) was used to measure aortic annular diameters and determine the correlation of its results with those derived from MDCT imaging.

### 3.3. Agreement of MDCT and 3D TEE Aortic Annular Diameter Measurements

**Figure 3:**
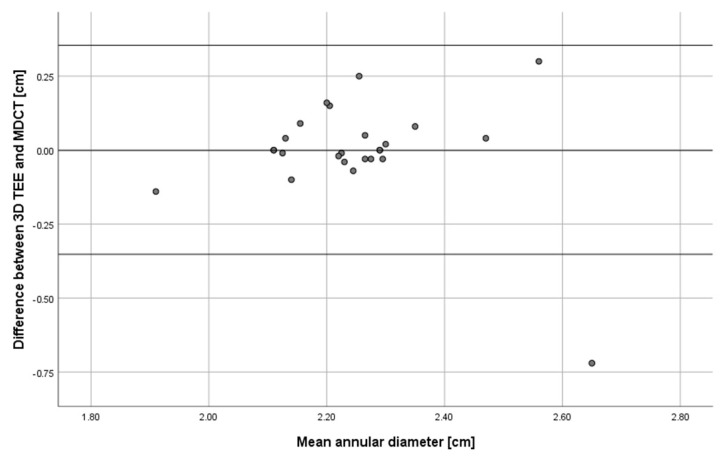
Bland-Altman plot showing agreement between TEE and MDCT measurements of aortic annulus diameter applied to images obtained from 25 patients.

There were only slight differences between annular diameter measurements obtained via MDCT (2.25 ± 0.19 cm) and 3D TEE (2.25 ± 0.15 cm). The mean difference between aortic annular diameter measured by MDCT and TEE was 0.001 cm. The Bland–Altman analysis indicated there was no proportional bias between 3D TEE and MDCT measurements. The agreement between the two measuring techniques and lack of proportional bias can be seen in a Bland–Altman plot (Figure 3).

### 3.4. Implantation of Prosthetic Valves

The most commonly used implant was the Edwards SAPIEN 3 26 mm valve (40%), followed by the Edwards SAPIEN 3 23 mm valve (20%) and the Edwards SAPIEN 3 29 mm valve (20%). MDCT values of the aortic annual diameter measurements were provided to the prosthetic valve manufacturers, as per their guidelines. The procedures were performed via the transfemoral approach in all the cases; therefore, the preprocedural protocol involved exploration of the entire aorta by imaging. Femoral access is predominantly used in TAVI because it is minimally invasive and allows the procedure to be performed in consciously sedated and locally anesthetized patients [12]. For the transfemoral approach, it is important to know the presence of pre-existing factors, such as abdominal aneurysm, aortic dissection, thrombosis, complex atheromas, and kinking of the aorta [13,14].

The 30-day mortality of the study population was 4%, represented by one intrahospital death subsequent to severe acute ischemia due to the prosthetic valve blocking the left iliac common artery.

## 4. Discussion

The main purpose of this study was to determine whether 3D TEE can be used interchangeably with MDCT as a preprocedural aortic annular diameter measuring technique for TAVI. Our results show that the 3D TEE aortic annular diameter measurements obtained could have been used interchangeably with the measurements obtained through the MDCT imaging technique for sizing of the Edwards SAPIEN and Medtronic CoreValve prosthetic aortic valves. Therefore, these results show that 3D TEE can be successfully employed for pre-TAVI aortic annular measurements when CT is unavailable or contraindicated to patients.

Preprocedural cardiac CT performed before TAVI has been shown to provide essential information about the aortic root and the peripheral access vessels by analyzing transaxial images, multiplanar and curved multiplanar reconstructions, maximum intensity projections, and 3D volume renderings [15]. MDCT evaluation of the whole aorta can detect multiple pre-existing factors that can impact the transfemoral approach used in TAVI and, therefore, should be used for the analysis of the aorta in case the transfemoral approach is employed, with transaxial images and multiplanar reconstructions being the most common techniques [13]. Furthermore, pre-TAVI low-dose low-contrast CT angiography evaluation has been shown to have a very good agreement with the standard of reference measurements and a quick reading time [16]. Moreover, a low contrast CT protocol using less than half of the amount of contrast agent usually used in the literature (38 mL vs. 80–120 mL) has been shown to be feasible for preprocedural TAVI planning [17]. A low contrast CT protocol can be clinically useful for patients with pre-existing conditions such as renal disease and diabetes because it can reduce the risk of renal dysfunction caused by the contrast agent.

However, 3D TEE has been shown to be a viable alternative to MDCT due to its strong correlation and/or agreement with MDCT aortic annular measurements [18,19,20,21], attesting its reliability in clinical settings and, therefore, its usefulness in providing correct measurements of the aortic annulus prior to TAVI, which is in concordance with the results of our study. The 3D TEE technique can also offer important information about aortic root morphology [22], such as different transverse areas and longitudinal distances between tricuspid and bicuspid AS patients, the latter being larger, entailing different impacts on pressure recovery. Therefore, 3D TEE is considered the ideal technique for pre-TAVI initial evaluation because it offers detailed information about the morphology and function of the left ventricle and aortic valve. In our initial evaluation of the 25 patients, 3D TEE was performed for the descending aorta and none of the patients presented aortic tortuosity or any other anomalies that could have prevented access to the vascular route. Furthermore, 3D TEE provides the possibility of assessing aortic valve area regardless of aortic valve morphology, as opposed to MDCT measurements, which were affected by the aortic valve morphology in patients with AS [23]. Compared to other imaging systems, such as MDCT or MRI, 3D TEE systems, alongside other US devices, are more cost-effective, have the advantage of portability, and can be successfully employed in patient bedside monitoring, increasing the quantity and quality of measurements and significantly benefiting patients that are intubated or can only receive bedside treatment [24].Therefore, we speculate that further studies and improvement of training for professionals, such as anesthesiologists and cardiologists, can result in increased TEE measuring accuracy and decreased time to conduct the procedure. Moreover, the 3D TEE method does not entail exposure to harmful contrast agents [25] and can be recommended for patients with dementia [26], renal failure [4], and other pathologies where MDCT is contraindicated or unavailable [19,27].

A recent study has shown that MDCT is more likely to lead to prosthesis oversizing than 3D TEE and that the risk of postoperative paravalvular regurgitation is decreased when both measurement methods are in agreement [28]. Therefore, 3D TEE may serve not only as an alternative to MDCT [29,30], but also as an important additional measuring technique where MDCT is available and indicated. Further research is required for the clinical usefulness of complementary 3D TEE measurements to MDCT in the sizing of prosthetic aortic valves used in TAVI and, therefore, in lowering the risk of paravalvular leakage. However, at the moment, there are several challenges facing 3D TEE, such as variability in measuring due to the experience of the operator or maintaining the position of the TEE probe transducer [31].

The development of novel software for analyzing TEE images has a profound impact on clinical outcomes, and it has been shown that both automated and semi-automated software can considerably reduce the time required for analysis and produce results that are in very good agreement with manually obtained values [20,32]. This can lead to more widespread adoption of TEE as a preprocedural aortic annular measuring technique for TAVI. The application of machine learning, a subfield of artificial intelligence that uses complex computational algorithms, in novel measurement analysis software can be especially useful in echocardiography as it would allow the fast analysis of large amounts of data and prediction of outcomes with high accuracy, while the software would be able to constantly improve the quality of its predictions with each new set of acquired data [33]. Therefore, artificial intelligence holds much promise for clinical practice and has already been demonstrated to be increasingly useful to the medical field, having the potential to improve the accuracy and considerably reduce the duration of the echocardiographic analysis.

### Complications

Recent studies that compare the TAVI and surgical aortic valve replacement procedures have focused on the neurological complications secondary to the replacement of the aortic valve, and, in a recent randomized study that evaluated the risk of cerebrovascular accidents for patients undergoing either TAVI or the conventional surgical procedure of valve replacement, it was found that both procedures posed a similar risk to patients [34]. For postprocedural care, all the patients involved in this study were observed in the intensive care unit and dual antiplatelet therapy was continued. A total of two patients underwent postprocedural inguinal hematoma and, in one patient, the valve was blocked in the iliac artery, leading to severe acute ischemia.

The small number of patients enrolled represents the main limitation of this study. Another limitation to our study may be due to the fact that TEE measurements were performed by a single expert, and results may vary depending on the experience of the operator.

## 5. Conclusions

TAVI is an alternative technique for treating severe AS in high-risk patients that are nonsurgical, and it may be associated with a low complication rate. 3D TEE aortic annular measurements using novel image generation and analysis software were in good agreement with MDCT measurements and, therefore, 3D TEE may be employed as an alternative to MDCT.

## Data Availability

The data presented in this study are available on request from the corresponding author. The data are not publicly available in order to limit the amount of publically-available patient personal information, as classified by the European Union General Data Protection Regulation.
